# Criteria for reporting incidental findings in clinical exome sequencing – a focus group study on professional practices and perspectives in Belgian genetic centres

**DOI:** 10.1186/s12920-019-0561-0

**Published:** 2019-08-20

**Authors:** Marlies Saelaert, Heidi Mertes, Tania Moerenhout, Elfride De Baere, Ignaas Devisch

**Affiliations:** 1 Department of Public Health and Primary Care, Philosophy of Medicine and Ethics Research Group, Ghent University, Campus Heymans (UZ Gent), Corneel Heymanslaan 10 – Building 6K3, 9000 Ghent, Belgium; 2 Department of Philosophy and Moral Sciences, Bioethics Institute Ghent, Ghent University, Ghent, Belgium; 3 Department of Philosophy and Moral Sciences, Ghent University, Ghent, Belgium; 4 Center for Medical Genetics Ghent (CMGG), Ghent University and Ghent University Hospital, Ghent, Belgium

**Keywords:** Incidental findings, Secondary findings, Clinical exome sequencing, Disclosure, Professional practice, Focus groups, Qualitative research

## Abstract

**Background:**

Incidental and secondary findings (IFs and SFs) are subject to ongoing discussion as potential consequences of clinical exome sequencing (ES). International policy documents vary on the reporting of these findings. Discussion points include the practice of unintentionally identified IFs versus deliberately pursued SFs, patient opt-out possibilities and the spectrum of reportable findings. The heterogeneity of advice permits a non-standardised disclosure but research is lacking on actual reporting practices. Therefore, this study assessed national reporting practices for IFs and SFs in clinical ES and the underlying professional perspectives.

**Methods:**

A qualitative focus group study has been undertaken, including professionals from Belgian centres for medical genetics (CMGs). Data were analysed thematically.

**Results:**

All Belgian CMGs participated in this study. Data analysis resulted in six main themes, including one regarding the reporting criteria used for IFs. All CMGs currently use ES-based panel testing. They have limited experience with IFs in clinical ES and are cautious about the pursuit of SFs. Two main reporting criteria for IFs were referred to by all CMGs: the clinical significance of the IF (including pathogenicity and medical actionability) and patient-related factors (including the patient’s preference to know and patient characteristics). The consensus over the importance of these criteria contrasted with their challenging interpretation and application. Points of concern included IFs’ pathogenicity in non-symptomatic persons, IFs concerning variants of uncertain significance, the requirement and definition of medical actionability and patient opt-out possibilities. Finally, reporting decisions were guided by the interaction between the clinical significance of the IF and patient characteristics. This interaction questions the possible disclosure of findings with context-dependent and personal utility, such as IFs concerning a carrier status. To evaluate the IF’s final relevance, a professional and case-by-case deliberation was considered essential.

**Conclusions:**

The challenging application of reporting criteria for IFs results in diversified practices and policy perspectives within Belgian CMGs. This echoes international concerns and may have consequences for effective policy recommendations.

## Background

Incidental findings (IFs) and secondary findings (SFs), which are variants in known disease genes unrelated to the diagnostic indication, are subject to ongoing discussion as potential consequences of clinical exome sequencing (ES) [1–3]. Since ES simultaneously covers all coding regions of a patient’s genome, results unrelated to the diagnostic question can be found unintentionally, as IFs, or deliberately pursued, as SFs [2–5]. As ES is increasingly implemented for the diagnosis of monogenic diseases, various policy documents have been published regarding IFs and SFs in the US, Europe and Canada [1–3, 5–8]. However, these documents differ on fundamental issues and none of them is accepted as the general standard. Issues regarding (i) a practice of unintentional IFs versus actively pursued SFs, (ii) patient opt-out possibilities and (iii) the spectrum of reportable findings remain unresolved [9–11].

Firstly, the American College of Medical Genetics and Genomics (ACMG) has published highly influential recommendations which advocate the routine analysis of an additional panel of 59 genes and the reporting of all (likely) pathogenic variants when performing clinical ES [2, 3]. Pathogenic (class 5) and likely pathogenic (class 4) variants can provide adequate grounds for altering a patient’s surveillance or treatment [12]. Class 3 variants of uncertain significance (VUS), however, should not be considered as sufficient grounds for clinical decision-making [12]. Even though VUS might be reported when possibly relevant to the diagnostic question, their reporting is not advised when identified as IFs [3, 4, 7, 8]. According to the ACMG, screening for (likely) pathogenic variants in the diagnostically unrelated gene panel should occur in every case of clinical exome and genome sequencing, as a realisation of the professional duty to avoid harm [2, 3, 10, 13]. However, this opportunistic screening has been criticised and the American Presidential Commission for the Study of Bioethical Issues (Bioethics Commission) notes how it might entail additional health risks, overwhelm patients with (ambivalent) information and stimulate a trend of medicalisation [5]. Therefore, the Bioethics Commission, and also EuroGentest, the European Society of Human Genetics (ESHG) and the Canadian College of Medical Geneticists (CCMG), are more cautious in their guidelines about reporting SFs and IFs [1, 5, 7, 8]. They advocate a strictly necessary and proportional application of ES and, if possible, (exome or genome-based) targeted panel testing, which only analyses a subset of known disease-associated genes and hence minimises the possibility of diagnostically unrelated IFs [1, 4, 7].

Secondly, the ACMG claims a patient’s right to opt out of deliberately pursued SFs [13]. Taking into account all other international policy documents’ advice of targeted testing and their restraint towards SFs, wide agreement on this opt-out possibility might be assumed [1, 7, 8]. International statements are more vague, however, about opting out of unintentional IFs. The Bioethics Commission, ESHG and Public Health Genetics (PHG) Foundation recommend that professionals should make a “prudent professional judgement” [5] concerning their fiduciary duty when a patient wants to opt out of an IF that is relevant, serious, and medically actionable (i.e. enabling surveillance and preventive and/or therapeutic interventions) [1, 3, 5, 14]. This way, a patient’s right not to know might be overruled by a professional’s presumed duty to avoid harm [3]. Recently published points to consider for laboratories, however, as well as the Canadian geneticists’ position statement, strongly advocate respect for a patient’s choice not to know IFs [6, 8].

Thirdly, the specific spectrum of genes or conditions that should be considered as reportable IFs or SFs, as well as the underlying reporting criteria, are strongly debated [15, 16]. Lists of conditions and associated genes (including the ACMG gene list) have been challenged by the critique that variants might be classified differently or might be less penetrant and expressive in asymptomatic persons [15]. Hence the identification of IFs or SFs as predictive disease risks might be doubted [17]. Finally, the possibility of medical actionability has been stressed as an important criterion for reportable IFs and SFs in various recommendations [1–3, 5]. Even though a semiquantitative metric has been developed as an attempt to assess medical actionability objectively, this criterion has been criticised [10, 18]. On the one hand, the mere availability of a medical intervention does not guarantee its effectiveness, and many interventions for conditions on the ACMG list are not supported in terms of their effectiveness by clinical trials or professional guidelines [14]. On the other hand, it has been suggested that the definition of medical actionability is too narrow and should also include reproductive choices or should be complemented by the criterion of personal utility (i.e. a personal interest or benefit that goes beyond improved healthcare outcomes) [15, 19].

Recently, the persistent lack of accord among policy documents has been exemplified in an international comparison of consent forms used for large gene panels, exome or genome sequencing. About half of the studied forms did not indicate their policy on reporting IFs or SFs and many used undefined terms (leaving the reference to IFs and/or SFs and corresponding reporting practices unclear) [20]. Moreover, the spectrum of reportable IFs and SFs (if specified) as well as the options to opt in for or opt out of (specific categories of) findings widely varied [20].

The diverse character of recommendations and consent forms and their inclusion of contested terms and criteria permits a non-standardised practice regarding IFs and SFs. However, only a limited amount of research has investigated the actual uptake of policy guidelines regarding IFs and SFs and has focussed on current reporting practices in a context of clinical ES. A US-based survey identified diverse practices regarding the spectrum of reportable IFs and SFs (which considerably exceeded the ACMG list) and different opt-in and opt-out possibilities [21]. Outside the US, two studies, each including laboratories from various countries, have analogously reported a variety in reported IFs [22, 23]. This study aims to further assess the actual practice regarding IFs and SFs in clinical ES, as well as to investigate the underlying professional perspectives. This research will also indicate which elements of international policy documents have been incorporated in practice as being most relevant or feasible and which elements demand further consideration or adjustment for efficient and successful policymaking.

## Methods

### Recruitment and data collection

To achieve an in-depth understanding of the practice and policy regarding IFs and SFs, a qualitative study was set up in Belgian centres for medical genetics (CMG). Belgium has eight CMGs: three in the Flemish Region, two in the Walloon Region and three in the Brussels-Capital Region. Since the aim was not to find out individual or role-specific views but the integrated perspective of each CMG, and to stimulate open conversation and interaction between colleagues, one focus group in every CMG was considered to be most appropriate [24]. A purposive sampling approach was used to recruit a multi-disciplinary and representative group of participants in every CMG, including both clinical and clinical laboratory geneticists and possibly other professionals. CMGs were informed about our study and its procedure by a presentation at the Belgian College of Medical Genetics (a federal body for quality of healthcare in medical genetics). Subsequently, a contact (usually the head of department) at each CMG was approached by email or telephone to request participation. If the contact agreed, they suggested a time which suited most of the CMG’s professionals.

All focus groups were conducted in a room at the CMG or associated hospital between November 2016 and December 2017, and lasted between 67 and 117 min. All focus groups were moderated by the first author and an observer was present and took field notes in seven out of eight focus groups. Focus groups were moderated in Dutch or English and participants could choose to speak Dutch, French or English.

A semi-structured interview guide, created after a thorough literature review, was evaluated by a multidisciplinary team of an ethicist (HM), geneticist (EDB) and philosopher (ID) and was used for all focus groups. Open-ended questions and probes to stimulate discussion were used (Table 1). Terminologically, “IF” was used to refer to unintentionally identified, diagnostically unrelated results. “SF” was used to refer to deliberately pursued, diagnostically unrelated findings. The study’s specific focus on IFs and SFs in clinical ES for monogenic diseases, excluding preconception, prenatal, screening and research contexts, was emphasised at the outset of every focus group.

**Table 1:** Examples of interview questions

How do you describe an IF in a clinical context in your CMG, apart from following guidelines? What terminology do you use?	
What differences do you see between IFs in array testing and in clinical ES?	
What kind of IFs do you report, firstly from the laboratory to the clinician, and secondly from the clinician to the patient?	
What kind of policy regarding IFs would you like to create in the future?	
What impact do international guidelines on reporting IFs have on your own practice?	
What difficulties do you experience in your practice regarding IFs or SFs? What are the great challenges in the evolution of IFs?	
What is your current practice regarding a patient’s request to opt out of IFs?	
How do you consider the intentional search for SFs?	
What is your practice when new information is available about an IF, for example for recontacting patients?	

Abbreviations used: *CMG* Centre for medical genetics, *IF(s)* Incidental finding(s), *SF(s)* Secondary finding(s), *ES* Exome sequencing.

### Data analysis

Focus groups were audio-recorded and transcribed verbatim and data are saved until completion of the full research project on a password-protected server. Data were analysed thematically, with an inductive approach and unrestricted by theoretical concepts. The analysis consisted of the consecutive stages of data immersion, code generation, theme identification, theme revision, theme definitions and production of the final report, as described by Braun and Clarke [25]. All data were coded by MS, and TM independently coded a substantial subset of the data. Analysis was an iterative and ongoing process during data collection. Text units could be included in more than one code and/or theme and the analysis was supported by use of a software program for qualitative data analysis (NVivo12). During analysis, ideas and reflections were stored as memos. An extensive procedure was developed to ensure the trustworthiness and credibility of the data collection, analysis and report. The procedure combined peer debriefing and a systematic audit trail, and covered both the process and the product of the analysis [26]. Following TM’s secondary analysis of a data subset, the transcripts and initial code schemes were reviewed and theme names, definitions and structures were thoroughly discussed by MS and TM. Preliminary thematic structures and draft reports were discussed exhaustively and reviewed by the multidisciplinary group of authors until consensus was reached between all of them. Finally, quotes were selected and, if originally in Dutch or French, translated by MS and TM to illustrate the results. This article adheres to the COREQ guidelines for reporting qualitative research [27].

## Results

All eight Belgian CMGs agreed to participate. Every focus group was composed multi-disciplinarily and involved between 6 and 11 participants, with a total number of 68 participating professionals (Table 2).

**Table 2:** Focus group participants

	FG1	FG2	FG3	FG4	FG5	FG6	FG7	FG8	Total
Participant’s profession									
Clinical geneticist	3	3	4	5	3	3	2	2	25
Clinical laboratory geneticist	3	3	4	2	4	2	2	6	26
Genetic counsellor/Psychologist		4	1	2	1	1	2		11
Other (Bio-informatician, Bioethicist, Trainee MD)		1		1			3	1	6
Total	6	11	9	10	8	6	9	9	68

Abbreviations used: *FG* Focus group.

Six themes emerged from the data analysis: (i) current and general practice in clinical genetic testing, (ii) the position of genetics in medicine and society, (iii) criteria for reporting IFs, (iv) impact of IFs and SFs, (v) policy guidelines for genetic practice, (vi) guiding values and principles. This article addresses the third theme of the reporting criteria for IFs in a context of clinical ES in adults.

When considering the reporting of IFs, Belgian CMGs referred to two major criteria: the clinical significance of the IF and patient-related factors.

### Clinical significance of the IF

Currently, Belgian CMGs do not analyse the full exome in clinical ES and mainly use exome-based panels, hitherto resulting in a rather limited experience with IFs in clinical ES. However, whole exome sequencing (WES) was identified as the undeniable future of clinical genetics. Due to the many monogenic conditions, IFs are expected to be frequent when very large panels or even the full exome will be analysed.

Professionals also referred to the possibility of screening additional genes as SFs when sequencing the exome, but a lack of (human, financial, and technical) resources and an unfulfilled need for guidelines (for example regarding reimbursement and the scope of analysis) fail to guarantee the required depth and trustworthiness of additional analyses in clinical WES. This could result in unnecessary interventions or harm and a false sense of security. Therefore, Belgian CMGs do not deliberately pursue SFs and only consider diagnostically unrelated findings in clinical ES when they are unintentionally identified as IFs (Table 3, Quote 1, Quote 2).

**Table 3:** Quotes

Number	Quote	Participant
Quote 1	*“Maybe, at random, we could find something and when we find something that we are sure of, we will tell you. [… ] But we won’t actively look for it.”*	FG 8 - P9 Clinical laboratory geneticist
Quote 2	*“There is a filter in accordance with the ACMG recommendations, but it is not used as standard. [ … ] It takes considerable human capacity to analyse those things and currently it is not included in our routine-protocol, to look at those things as standard.”*	FG 7 - P7 Clinical laboratory geneticist
Quote 3	*“The reporting of variants where even we don’t know whether they mean anything, is the equivalent to reporting non-information which might make a patient despair or ask for an impossible follow-up. [… ] So I think we have a responsibility as professionals not to go that far.”*	FG 2 - P10 Clinical laboratory geneticist
Quote 4	*A: “The example would be, in theory, because now we wouldn’t see it, eh, Huntington’s disease, if you see that, at whatever age, should you transmit [report] it? So far, the answer is no. [… ] And any other change for which you have nothing to offer to the patient, we don’t report.” […]* *B: “But you can have Parkinson’s, Alzheimer’s … with a point mutation, for example.”* *A:*	FG 1 A = P5, Clinical geneticist B = P1, Clinical geneticist
Quote 5	*“[… ] and then, during the next pregnancy, they would find out that their child has Duchenne … You don’t want to have this [kind of situation], whereas we have seen it during a previous [test], for example in their daughter. So currently, we don’t work with an opt-out, to avoid this kind of thing. And I’ve never met a family who had problems with this [practice].”*	FG 2 - P2 Clinical geneticist
Quote 6	*“[… ] so people have to decide between opt-in and opt-out, and I think it is very complex for people to be sufficiently informed about this choice. [… ] It’s hopeless, people can’t choose, well, most of them can’t. I mean, most people don’t have any background knowledge of genetics, so it is extremely difficult.”*	FG 4 - P7 Psychologist
Quote 7	*“A possibility to choose … You cannot simply force someone, I mean, you can’t just … The aim of informed consent is to make a deliberate choice, so you have to give people the right information, so they can make a choice. I think that is the aim of informed consent.”*	FG 6 - P1 Genetic counsellor
Quote 8	A: ***“****But, perhaps I don’t understand, if the patient chooses [an] opt-out, then we don’t report. [… ]* ***I****f the patient chooses, and it’s clear, to opt out, then we don’t report.”*	FG 3 A = P1, Clinical geneticist
	B: *“But I would anyway! [… ] I would say, the consequence of not reporting and maybe losing a parent, for example the [patient’s] mother very, very young … Those consequences are so important that I would choose to disregard the patient’s decision, I would note it down in the file that I choose to disregard, because [… ] I consider the consequence of not reporting to be worse than the consequences of reporting.”*	B = P8, Clinical geneticist
Quote 9	*“So generally, when we have an IF like that, most of the time there is a deliberation between the biologist [clinical laboratory geneticist] who is responsible for the analysis and the clinician [clinical geneticist] who validates the test. [… ] Sometimes, we even call upon external people, other centres, or people who have the right experience.”*	FG 5 - P5 Clinical geneticist

Abbreviations used: *FG* Focus group, *P* Participant, *IF(s)* Incidental finding(s), *ACMG* American College of Medical Genetics and Genomics

According to professionals in Belgian CMGs, reported IFs should be clinically significant, i.e. they should be relevant to a patient’s health. CMGs especially referred to pathogenicity and medical actionability as important components of an IF’s clinical significance. However, throughout the focus groups, the exact delineation of these criteria and their application in practice has turned out to be challenging.

#### Pathogenicity

A reported IF has to be a clinical risk factor, i.e. a variant predicted to cause disease, and various CMGs apply and advocated a cut-off for pathogenicity in reportable IFs. They suggested only reporting class 5 (pathogenic) and class 4 (likely pathogenic) variants in diagnostically unrelated but known disease genes. Class 3 variants (variants of uncertain significance or VUS), for example in an unrelated breast cancer gene, are not reported, as this might have a significant psychological impact or, as a consequence of unnecessary interventions, medically harmful consequences (Quote 3).

However, several factors complicate the definition of clearly pathogenic IFs. Firstly, verifying IFs’ pathogenicity and predictive value in any particular patient is challenging in general, as there is usually no corresponding phenotype (i.e. patients are non-symptomatic for the IF’s associated disease). Secondly, professionals described how, in the future, the advice not to report VUS in IFs might not always be realised. When a VUS is identified, it can be difficult to determine whether the affected gene is related to the symptomatic condition or not. CMGs noted that a VUS in a diagnostically relevant gene is sometimes reported, but when the gene’s diagnostic relevance is not fully guaranteed, this reporting might undermine the cut-off for pathogenicity in IFs. Thirdly, variant classifications are dynamic and a VUS may be reclassified as a pathogenic variant over time. When this variant turns out to be relevant to the symptomatic condition, its reclassification may eventually lead to a diagnosis, which patients usually experience as a relief. Therefore, professionals acknowledged the duty to recontact patients regarding the reinterpretation of diagnostic results. In the context of IFs, however, recontacting patients regarding a reclassified VUS, was regarded as logistically impossible. Moreover, professionals suggested that such a delayed report of an IF would only be appropriate if patients explicitly agreed to it, as this finding is not directly related to the indication for testing and does not realise the pursued diagnosis.

#### Medical actionability

Most CMGs exclusively report actionable IFs, which were described as findings for which medical therapy, treatment or preventive screening are available (Quote 4). Several professionals regarded the knowledge of IFs ad infinitum, including non-actionable IFs, as harmful, because this includes information that patients do not understand and cannot handle (practically or psychologically). Professionals also expressed feeling powerless themselves about non-actionable IFs and feeling “more comfortable with a cancer predisposition than with [a predisposition for] a neuro-degenerative condition”. These professionals considered the limitation of reportable IFs to actionable results as a consequence of their professional duty and responsibility to decide on relevant information. Ultimately, only reporting actionable IFs was presented as a pragmatic way of keeping clinical ES practically feasible, as excluding non-actionable findings reduces the time required for analysis.

Even though they had not actually been in this situation, some professionals remarked that not reporting non-actionable IFs, for example regarding a neuro-degenerative condition, could be an ethically difficult decision, as it would withhold important information from patients and/or their families. Therefore, one CMG explicitly stated that if they identified serious, non-actionable IFs, these would be reported. Another CMG suggested that non-actionable results might, depending on the specific circumstances, be reported as IFs, but, if a practice for SFs were developed, these deliberately pursued results should only concern medically actionable findings.

The use of a standard list of medically actionable genes was proposed. Many Belgian CMGs use the ACMG list of “highly penetrant and actionable genes” as a (not strictly binding) framework for reportable IFs [2, 3]. On the other hand, some CMGs considered such a list as being in conflict with the dynamic reality of treatments and preventions that can become available over time. Hence a variant’s actionability might better be determined at the time of discovery.

Finally, a correlation was suggested between a condition’s actionability and penetrance. Even though risks are subjectively interpreted, “low penetrance” IFs were considered to be too abstract, and classifying them as actionable might create unrealistic expectations regarding the utility of this information. Therefore, as a suggestion for future policy, actionable IFs should be highly penetrant and patients should be counselled in interpreting incomplete penetrance.

### Patient-related factors

As a second criterion for reporting IFs, CMGs referred to patient-related factors, being the patient’s preference to know IFs and patient characteristics.

#### Preference to know

ES allows a selection of analysed genes and hence, theoretically, a choice to receive IFs or not. However, not all Belgian CMGs offer this opportunity, and practices on a patient opt-in and opt-out vary widely.

Three CMGs currently offer no opt-out of actionable IFs, although one of them offers an opt-out of non-actionable IFs. Professionals at these CMGs argued firstly that they have the ambition or even the duty to prevent future disease that can be avoided (Quote 5) and secondly that lay people do not truly understand the meaning and possible impact of IFs. In the event of an opt-out, patients would not realise what they are actually declining (Quote 6). As a third argument, two CMGs mentioned their ethics committee’s influence on this policy. It did not allow an opt-out of actionable IFs because professionals should report useful information when it is available and, again, because patients would not understand their own decision. Finally, it was observed that patients generally do not dispute the possibility of receiving actionable IFs. One CMG added that, even if an opt-out is not suggested, patients can spontaneously ask for it, but no such cases were mentioned during the focus group.

Despite these arguments, one CMG explicitly discussed its current policy and reported an exception by honouring a patient’s request not to look at breast cancer genes during an unrelated clinical ES. Two centres mentioned that it would be good to update their ethics committee on recent developments in clinical ES, possibly to re-evaluate their opt-out policy. Finally, it was recognised that patients might be distressed when discovering future health risks as IFs. Nonetheless, these worries were said to be inevitable, as the risk would probably manifest itself anyway at a later point in life.

Conversely, four CMGs always allow an opt-out of actionable IFs. To justify their policy, these CMGs also referred to the idea that IFs might be complex to comprehend, also in psychological terms. Some patients might not be able to deal with the information, and therefore their preference to opt out of these results should be respected. Moreover, these CMGs stated that patients’ general and fundamental right not to know should be honoured (Quote 7). Nevertheless, it was mentioned that only a small minority of patients actually choose to opt out of actionable IFs.

Finally, one CMG, with limited experience with clinical ES, discussed its future policy and the possibility of an opt-out in depth. While some of its professionals strongly defended absolute respect for a patient’s choice, one participant claimed that opting out should only be accepted if it has minor implications for the patient’s prognosis. When not reporting actionable IFs could have severe consequences, this professional would overrule a patient’s opt-out. Again it was argued that patients do not understand what IFs and an opt-out really mean. Moreover, the possible harm of not reporting an actionable IF would outweigh the harm of being informed against one’s will (Quote 8). To reconcile the two perspectives in this CMG, two opposing solutions were suggested for a patient’s opt-out: the IF could be reported at a later and more suitable moment, or the IF could be masked in the report from the laboratory to the clinician. That way, situations where the clinician knows but cannot disclose relevant patient information could be avoided.

#### Patient characteristics

Finally, professionals noted that patient characteristics influence whether and how an IF is reported, as patients' (clinical and personal) context interacts with the IF’s clinical significance and affects its final relevance. Professionals provided the hypothetical example of the importance of a patient’s primary condition for the timing of reporting an IF as well as for the suggested follow-up and counselling, since both results are considered to have an integrated impact on a patient’s health and life. A patient’s wish for future children or his/her family history of illness might also affect the disclosure of an IF regarding a carrier status or of a non-actionable IF (since it could explain an undiagnosed family condition). These last examples illustrated how personal and family characteristics interact with the definition of actionability and hence might affect an IF’s clinical significance and disclosure. Most CMGs did not consider actionability to include lifestyle adjustments or personally useful actions. However, they did discuss actionability in terms of reproductive decision making, which would enable the reporting of IFs concerning a carrier status for a recessive condition. Some professionals do or would not report these findings, because they are not clinically threatening for patients themselves. It was also mentioned that including prenatal possibilities “would make every condition actionable”. However, and depending on personal and/or family characteristics and plans, these results can be relevant to relatives and (future) children. Therefore, half of the CMGs would consider the reporting of IFs regarding a carrier status for severe diseases (for example cystic fibrosis or Duchenne muscular dystrophy). Two centres already reported such findings and one does not offer an opt-out of them. Nonetheless, the psychological impact of this disclosure was acknowledged and one CMG testified about a family that was emotionally upset by the disclosure of a cystic fibrosis carrier status.

The interaction between, on the one hand, a patient’s characteristics and (clinical, personal, reproductive, family, etc.) context and, on the other hand, the clinical significance (including the actionability) of IFs does not result in a standard outcome, and hence evaluating an IF’s final relevance frequently requires a professional, multidisciplinary deliberation (Quote 9). To facilitate the deliberation process, a national (online) consortium on IFs was suggested, where “difficult cases” could be discussed, as well as a specialist committee to relieve CMGs of the exclusive responsibility regarding disclosure.

Despite the case-by-case deliberation, some professionals would still prefer general guidelines, for example regarding pathogenic variants and actionability, to facilitate the professional decision about disclosure.

## Discussion

An analysis of current practice at Belgian CMGs regarding clinical ES in the context of adult testing revealed a diagnostic focus and a standard procedure of exome-based panel testing, resulting in a low incidence of IFs. Belgian CMGs’ collective policy not to deliberately pursue SFs mirrors the avoidance of diagnostically unrelated findings and accords with current laboratory practices and with all international guidelines apart from the ACMG recommendations [1–3, 6–8, 23, 28].

Whether CMGs report an IF is determined by an interaction between the clinical significance of the IF and patient-related factors.

### Clinical significance of the IF

Belgian professionals indicated pathogenicity and medical actionability as important components of an IF’s clinical significance. These criteria are not surprising in themselves, as they are also stressed by leading American and European recommendations [1–3, 7]. Nonetheless, these criteria were extensively discussed because their interpretation and application in practice turns out to be challenging.

The importance of IFs’ pathogenicity was unanimously emphasised. However, Belgian CMGs also expressed concerns about IFs’ disease predictive value in asymptomatic persons. This idea is echoed internationally, even by the ACMG itself [3, 6, 9, 29, 30]. Richards et al. mentioned that variants might be less pathogenic and less penetrant if they are unrelated to the primary test indication and when there is no phenotype or family history of the associated condition [12]. The caution with which Belgian professionals approach the pathogenicity of IFs reflects these remarks, as well as the warning that unreliably interpreted and reported results might cause physical and psychological harm [15, 31]. The parallel idea expressed by Belgian CMGs regarding a cut-off for pathogenicity in IFs, and the suggestion to only report class 5 and class 4 but not class 3 variants (VUS), accords with international laboratory practices and points to consider, and with the ACMG recommendations [2–4, 6, 21]. Not reporting VUS in IFs from the laboratory to the ordering clinician prevents an over-interpretation of these results’ significance for the diagnostic question and needless patient follow-up [6, 23, 32].

Along with pathogenicity, most but not all CMGs assessed an additional threshold for reportable IFs, being their actionability. This criterion, as well as its interpretation as *medical* actionability, corresponds with an international consensus and might be partly explained by professionals’ specific role as medical experts [1, 3, 5, 11, 28–30, 33]. Nonetheless, the exclusive reporting of actionable IFs was characterised as a dynamic and ethically difficult policy by some Belgian centres. Some CMGs suggested to identify low penetrance IFs as non-actionable, which refers to a correlation between criteria that has already been indicated by the ACMG and its current list of 59 “highly penetrant and actionable genes” that should be analysed as SFs [2, 3]. Conversely, it has also been suggested that variants’ low penetrance can be countered by the associated condition’s actionability [28, 30].

### Patient-related factors

A notable finding of this study is Belgium’s diverse practice regarding the opt-out of actionable IFs. The absence of the possibility to opt out was legitimised by the professional aim to avoid harm. However, in-house discussions about this mandatory opt-in policy and professional concerns about the psychological impact of reported IFs illustrated that the superiority of professional duty over a patient’s choice is not self-evident. This value conflict was most visible in one CMG’s consideration of overruling a patient’s choice to opt out when it was considered to have harmful consequences. The denial of a patient’s preference, granting this criterion only a relative weight, sounds polemical but is in line with the “prudent professional judgement” which is advocated by bodies including the ESHG and Bioethics Commission [1, 5]. It also reflects the idea that the denial of a patient’s choice is sometimes inevitable, for example when a patient opts out of clinically relevant or medically actionable IFs [9, 16, 31]. The second argument for the obligatory disclosure of actionable IFs, being patients’ presumed inability to fully understand their impact, has also been suggested internationally [9, 32]. However, postulating a patient’s inability to make well-informed decisions might discount the efficacy of counselling procedures [5, 8, 34].

Patients’ general acceptance of disclosing actionable IFs supports the consolidation of offering no opt-out. However, an absence of questions might not necessarily equal an omnipresent preference to actually know IFs. When a CMG does not suggest an opt-out, few patients might have the genetic literacy to ask for one spontaneously, since the public understanding of genetics and its possibilities seems to be rather limited [35]. Moreover, it takes courage to dispute the professional authority of an informed consent form or pre-test counselling, or to resist the societal pressure to know as much as possible [33, 36]. Finally, a Belgian CMG’s claim that an IF, and the corresponding psychological distress, will manifest itself anyway at a later time might not be completely valid, as the incidentally identified variant could have an incomplete penetrance and/or variable expression, an idea related to the uncertain pathogenicity of IFs in asymptomatic persons.

Belgian CMGs that allow an opt-out of actionable IFs emphasise the honouring of patients’ wishes and their right not to know. This conflicts with well-known European recommendations but accords with a Canadian position statement and recent points to consider, and it is supported by international professional preferences [6, 8, 30, 37]. The Belgian suggestion that results should be masked in the laboratory report for the clinician when patients opt out, has been expressed internationally [31]. However, problems might arise when, as a result of changed circumstances or values, patients change their mind and do want to know IFs [16].

Finally, there is a general agreement, both within Belgian CMGs and internationally, that the interaction between an IF’s clinical significance and patient characteristics affects the final relevance of an IF [9, 15, 16, 23, 30, 32, 33]. This interaction clearly shows in the impact of a patient’s personal context on the criterion and definition of IFs' actionability and, more particularly, in the relevance and possible disclosure of IFs regarding a carrier status. Both within Belgian CMGs and internationally, this possible reporting is strongly discussed, as it might enable reproductive and/or (future) family-wide choices and actions [9, 23, 30, 33]. Even though reporting a carrier status for recessive conditions is in conflict with Belgian CMGs’ general focus on direct, medical actionability, half of them would favour such reporting to adults. This disclosure is supported by international professionals’ preferences and recent laboratory points to consider [6, 16, 21, 23]. On the other hand, it conflicts with the ACMG recommendations and creates an additional workload for results which are not clinically significant for patients themselves [2, 3, 16, 23]. However, this claim of reduced significance is countered by the impact of a reported carrier status on a person’s self-concept and specifically by the way it might threaten a person’s genetic identity, (future) health perception or wished-for parental role [38, 39], a psychological effect which was also insinuated by two Belgian CMGs. The impact of knowing one’s carrier status might even be more substantial in the case of a serious X-linked condition, such as fragile X syndrome, where carrying the premutation might also have clinical consequences for the carrier herself [40]. Belgian professionals did not raise this specific example of an IF regarding a carrier status, but nonetheless it goes against their statement that such a finding is non-threatening for patients themselves.

The reporting of IFs regarding a carrier status because of its possible value in the specific (personal or family) context of reproduction, can be considered in the more general debate on personal utility [19]. The concept of personal utility might, based on a patient’s characteristics and context, categorise findings which allow future (reproductive) choices, psychological or social coping or intrinsically valuable self-knowledge as reportable results, as they enable non-medical but valuable actions [19, 41]. Even though CMGs acknowledge the importance of a patient’s context and the difficulty of not reporting medically non-actionable IFs, most CMGs are not likely to add these options of personal utility to the actionability-criterion. Moreover, personal utility risks becoming an unspecified umbrella term that justifies the reporting of any kind of results [19, 42]. Therefore, Bunnik et al. suggest limiting personal utility to meaningful, technically and clinically valid information which “can reasonably be used for decisions, actions or self-understanding” [42]. As a consequence of its problematic definition, Vears et al. even suggest not assessing actionability as a decisive criterion for reporting pathogenically significant IFs [6].

As a second consequence of the interaction between an IF’s clinical significance and patient characteristics, the value of professional deliberation is stressed by both Belgian and international professionals [9, 11, 16, 23]. On the other hand, some CMGs’ call for guidelines on pathogenicity or actionability is also mirrored in international research, for example, concerning clinical laboratory geneticists who favour a list of conditions and genes that should be considered [9, 11, 15, 23, 29].

The tension between a call for (more) guidelines and a patient-specific, case-by-case deliberation has been identified previously [23]. As ES is increasingly implemented in clinical practice, it seems advisable, at least at a local level of CMGs, to create a guiding framework which is clarified to patients before testing and which relieves professionals from the responsibility to individually decide on every case of IFs. To further avoid the chance and injustice of offering different information to different patients, not only within but also between CMGs, an (inter)national consensus on relevant criteria might be pursued as a starting point for reporting practices [20, 22, 23, 43]. However, if general guidelines turn out to be unfeasible and the current diversity in national practice, as disclosed by this study, and in international practice and policy documents is maintained, a patient’s informed decision on which results to receive, starts with his/her choice of a specific CMG. In that case, it is quintessential for every CMG to disclose its local policy. On the other hand, and in line with the non-standardised outcome of the interaction between the clinical significance of IFs and patient characteristics, a flexibility in guidelines’ application has been advocated so they can be accustomed to the particular context [44]. Together with the professional expertise in CMGs, this call for a personalised deliberation nuances the need for and effectiveness of a rigid “one model fits all” policy [23]. Therefore, the contextualised application of a guiding framework of reporting criteria for IFs, might result in a personalised, non-standardised outcome.

To our knowledge, this is the first study on reporting practices and criteria regarding IFs which includes nationwide certified CMGs and hence achieves a good coverage of a national, non-commercial practice. The organization of one focus group in every centre revealed the similarities and differences in practice between centres. Moreover, it encouraged an open discussion between colleagues and a clarification of underlying reporting criteria. These results emerged from a Belgian context, with its specific scale and healthcare organisation. Nonetheless, the results of this study might be (partly) transferrable to other (and larger) countries with similar healthcare systems and analogous confrontations with diverse international guidelines, but further research is needed to confirm or deny similarities in practice and policy. As a consequence of Belgian CMGs’ standard practice of diagnostic exome-based panel testing, there is still a limited experience with actual IFs in clinical ES. Consequently, the perspectives expressed by the CMGs might reflect current reporting practices as well as preferable future policies. Future research should identify whether these perspectives are actually effected when the exome is fully analysed in clinical practice and IFs become more frequent.

## Conclusions

Belgian CMGs agree in their reference to common and internationally suggested reporting criteria for IFs. However, these criteria resist a uniform interpretation and hence result in a diversified Belgian practice, which reflects divergent, international policy perspectives. Belgian CMGs consent to the threshold of pathogenicity but concerns about IFs’ predictive value in non-symptomatic persons and VUS in IFs challenge this criterion’s application in practice. Furthermore, (medical) actionability is both an advocated and contested threshold, both internationally and at Belgian CMGs. In their adherence to international perspectives, Belgian CMGs differ most manifestly regarding patient opt-out possibilities for actionable IFs and in the weighing of professional duty versus patient autonomy. Finally, the interaction between the clinical significance of IFs and patient characteristics questions the definition of actionability and the possible reporting of IFs with personal utility such as findings concerning a carrier status. The importance of the patient’s context and the non-standardised outcome of its interaction with IFs’ clinical significance suggest the imminent inclusion of case-by-case reflections in reporting decisions. Accordingly, (international) guidelines for the reporting of IFs in clinical ES might only be effective when they are sufficiently detailed in terms of the criteria applied as well as responsive to the particularity of each individual case.

## Data Availability

None of the data generated and analysed during this study are publically available for reasons of personal privacy, but they are available from the corresponding author in response to a reasonable request.

## Abbreviations

ACMG: American College of Medical Genetics and Genomics
Bioethics Commission: American Presidential Commission for the Study of Bioethical Issues
CCMG: Canadian College of Medical Geneticists
CMG(s): Centre(s) for medical genetics
ES: Exome sequencing
ESHG: European Society of Human Genetics
FG: Focus group
IF(s): Incidental finding(s)
P: Participant
PHG Foundation: Public Health Genetics Foundation
SF(s): Secondary finding(s)
VUS: Variant(s) of uncertain significance
WES: Whole exome sequencing
